# Accurate evaluation of axillary sentinel lymph node metastasis using contrast-enhanced ultrasonography with Sonazoid in breast cancer: a preliminary clinical trial

**DOI:** 10.1186/s40064-015-1291-1

**Published:** 2015-09-17

**Authors:** Fumihiko Matsuzawa, Kiyoka Omoto, Takahiro Einama, Hironori Abe, Takashi Suzuki, Jun Hamaguchi, Terumi Kaga, Mami Sato, Masako Oomura, Yumiko Takata, Ayako Fujibe, Chie Takeda, Etsuya Tamura, Akinobu Taketomi, Kenichi Kyuno

**Affiliations:** Department of Surgery, Hokkaido Social Work Association Obihiro Hospital, 2 East 5 South 9, Obihiro, Hokkaido 080-0805 Japan; Department of Gastroenterological Surgery I, Hokkaido University Graduate School of Medicine, North 15, West 7, Kita-Ku, Sapporo, Hokkaido 060-8638 Japan; Diagnostic Ultrasound Division, Department of Laboratory Medicine, Saitama Medical Center, Jichi Medical University, 1-847 Amanuma-cho, Omiya-ku, Saitama, 330-8503 Japan; Department of Clinical Laboratory, Hokkaido Social Work Association Obihiro Hospital, 2 East 5 South 9, Obihiro, Hokkaido 080-0805 Japan

**Keywords:** Sonazoid, Breast cancer, Sentinel lymph node, Contrast enhanced ultrasonography, Axillary lymph node metastasis, Sentinel lymph node biopsy

## Abstract

Breast cancer is the most common type of cancer in women. The 5-year survival rate in patients with breast cancer ranges from 74 to 82 %. Sentinel lymph node biopsy has become an alternative to axillary lymph node dissection for nodal staging. We evaluated the detection of the sentinel lymph node and metastasis of the lymph node using contrast enhanced ultrasonography with Sonazoid. Between December 2013 and May 2014, 32 patients with operable breast cancer were enrolled in this study. We evaluated the detection of axillary sentinel lymph nodes and the evaluation of axillary lymph nodes metastasis using contrast enhanced computed tomography, color Doppler ultrasonography and contrast enhanced ultrasonography with Sonazoid. All the sentinel lymph nodes were identified, and the sentinel lymph nodes detected by contrast enhanced ultrasonography with Sonazoid corresponded with those detected by computed tomography lymphography and indigo carmine method. The detection of metastasis based on contrast enhanced computed tomography were sensitivity 20.0 %, specificity 88.2 %, PPV 60.0 %, NPV 55.6 %, accuracy 56.3 %. Based on color Doppler ultrasonography, the results were sensitivity 36.4 %, specificity 95.2 %, PPV 80.0 %, NPV 74.1 %, accuracy 75.0 %. Based on contrast enhanced ultrasonography with Sonazoid, the results were sensitivity 81.8 %, specificity 95.2 %, PPV 90.0 %, NPV 90.9 %, accuracy 90.6 %. The results suggested that contrast enhanced ultrasonography with Sonazoid was the most accurate among the evaluations of these modalities. In the future, we believe that our method would take the place of conventional sentinel lymph node biopsy for an axillary staging method.

## Background

For nodal staging of breast cancer, sentinel lymph node biopsy (SLNB) has become an alternative to axillary lymph node dissection (ALND) (Lyman et al. 2005; McCready et al. 2005). If the sentinel lymph node (SLN) is free of metastasis, it is not likely that metastatic disease is present in the axillary lymph nodes (ALNs); therefore, ALND can then be avoided (Dabakuyo et al. 2009).

Generally, SLNB for breast cancer is usually undertaken using dye and/or radioisotope labeling. However these methods have the problem, for example, high costs, requirement of a high skill (Yamamoto et al. 2015). Recently, a new method of SLN detection using contrast enhanced ultrasonography (CEUS) with Sonazoid was reported. The advantages of this method are this CEUS with Sonazoid method requires only a ultrasonography (US) apparatus and a contrast agent readily available on market and does not need radioactive materials (Omoto et al. 2009).

Metastatic lymph nodes exhibit peripheral and mixed vascularity because of the tumor angiogenesis. Color Doppler US can only provide information regarding the macro vessel flow and morphology; therefore, it is difficult to accurately diagnosis lymph node metastasis using this method (Yang et al. 2000). Sonazoid, a new generation contrast agent for US, allows for visualization of lymph node micro vessels. Compared with previously used imaging modalities, CEUS with Sonazoid would be expected to result in more accurate evaluation of lymph node metastasis.

In this study, we evaluated the usefulness of SLNs detection using CEUS with Sonazoid injected subareolarly and the presence of metastasis of SLNs using CEUS by intravenous injection of Sonazoid in breast cancer patients.

## Results and discussion

### Results

**Fig. 1:**
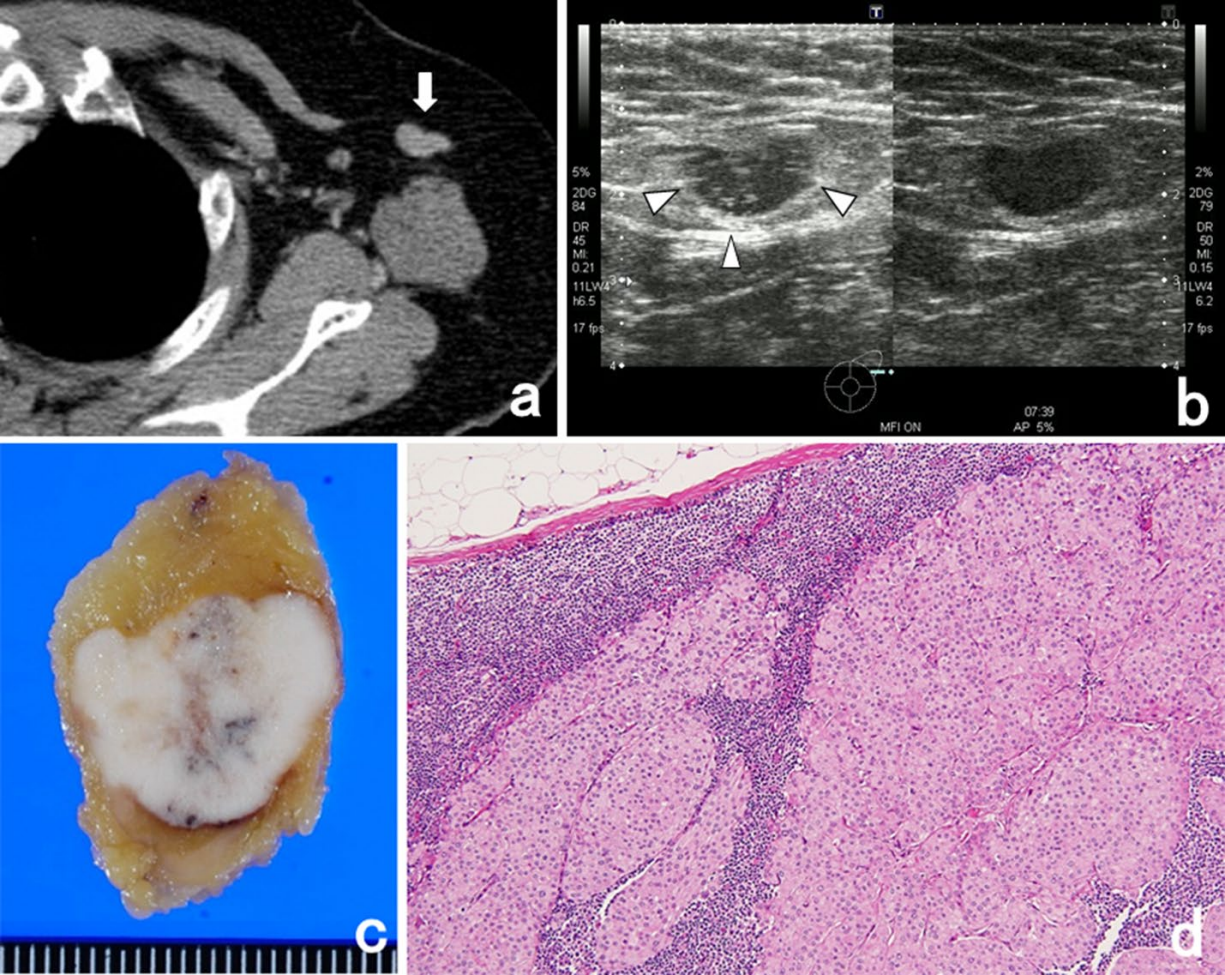
Representative images of a patient with pathologically positive metastasis of axillary lymph node. CECT images showed the non-enhanced axillary lymph node (**a**). CEUS imaging enhanced with Sonazoid revealed that there were the blood flows from multiple micro vessels from the other place of hilum (**b**). Pathological findings showed a metastasis of the axillary lymph node (**c**, **d**). *White arrow* shows the axillary sentinel lymph node. *White arrow heads* show the micro vessel from the other places of hilum

**Fig. 2:**
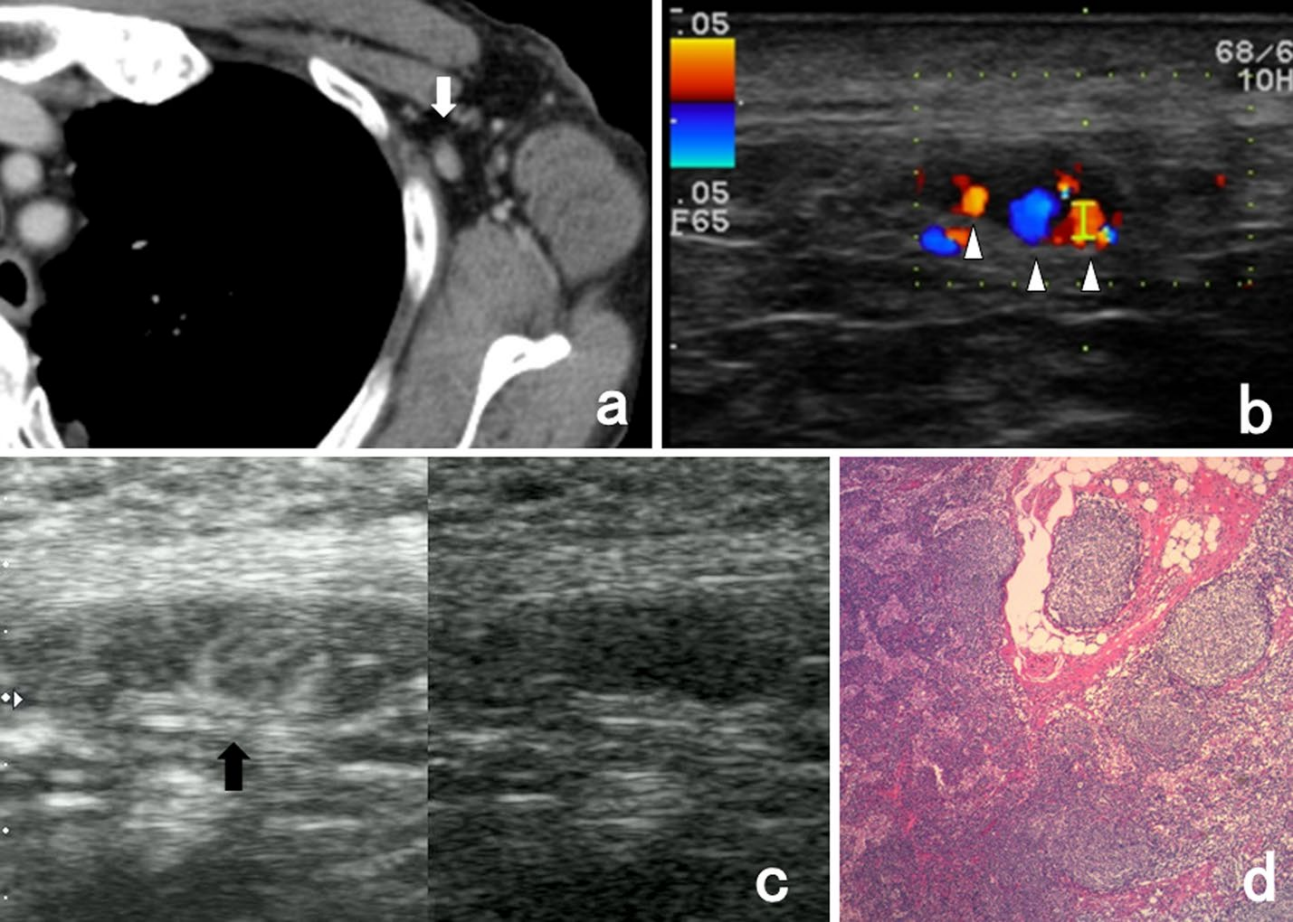
Representative images of a patient with pathologically negative metastasis of axillary lymph node. CECT images showed moderate-enhanced axillary lymph node (**a**). Color Doppler US showed blood flows from multiple micro vessel (**b**). CEUS imaging with Sonazoid revealed that these flows origin from a single vessel in the hilum (**c**). Pathological findings showed the negative metastasis of this axillary lymph node (**d**). *White arrow* shows the axillary sentinel lymph node. *White arrow heads* show the micro vessel from the other places of hilum. *Black arrow* shows the blood flows from a single micro vessel

CEUS with Sonazoid administering intravenously could visualize microvascularities in SLNs. Figure 1 showed the representative axillary lymph node images of positive metastasis. As shown in the figure, CEUS with Sonazoid administering intravenously revealed the multiple vascularity also from other places of hilum. On the other hand, Fig. 2 showed the representative images of negative metastasis. In the images of color Doppler US, there seemed to be several vascularities also from other places of hilum, but images of CEUS with Sonazoid administering intravenously revealed that these vasclarities flowed from only hilum of this lymph node.

### Identification of SLN using CEUS with Sonazoid administering subareolarly

All the SLNs were identified by using CEUS with Sonazoid administering subareolarly, and these SLNs corresponded with the SLNs detected by CT lymphography and indigo carmine method.

### Evaluation of axillary lymph node metastasis in SLNs by using CECT, color Doppler US and CEUS with Sonazoid administering intravenously compared with pathological findings

The results based on CECT were sensitivity 20.0 %, specificity 88.2 %, PPV 60.0 %, NPV 55.6 %, accuracy 56.3 %. Based on color Doppler US, the results were sensitivity 36.4 %, specificity 95.2 %, PPV 80.0 %, NPV 74.1 %, accuracy 75.0 %. Based on CEUS with Sonazoid administering intravenously, the results were sensitivity 81.8 %, specificity 95.2 %, PPV 90.0 %, NPV 90.9 %, accuracy 90.6 % (Table 1). These results showed that CEUS with Sonazoid was the most accurate among the evaluations of these modalities.

**Table 1:** Comparison of results of axillary lymph node metastasis detection by CT, Doppler US and CEUS with Sonazoid

	CECT	Color doppler US	CEUS with Sonazoid	CECT	Color doppler US	CEUS with Sonazoid
	–	+	–	+	–	+
Pathology						
–	15	2	20	1	20	1
+	12	3	7	4	2	9
Sensitivity (%)	20		36.4		81.8	
Specificity (%)	88.2		95.2		95.2	
PPV (%)	60		80		90	
NPV (%)	55.6		74.1		90.9	
Accuracy (%)	56.3		75		90.6	

*CECT* contrast enhanced computed tomography, *US* ultrasonography, *CEUS* contrast enhanced ultrasonography

## Discussion

Since standard radical mastectomy for the treatment of breast cancer was first established by Halsted, surgical procedures for breast cancer have continued to improve on the basis of the results of randomized clinical trials (McCready et al. 2005; Fisher et al. 2002; Jacobson et al. 1995; Lacour et al. 1983; Maddox et al. 1983; Turner et al. 1981).

Breast-conserving treatment is now regarded as a standard local treatment for early breast cancer. More recently, SLNB has become an alternative to ALND for nodal staging (Lyman et al. 2005; McCready et al. 2005). The SLN hypothesis states that tumor cells that are shed from a primary carcinoma migrate through a lymphatic channel to a single lymph node before involving further lymph nodes within that basin. The SLN is the first lymph node that receives lymphatic drainage from a tumor, and its identification and analysis for tumor involvement can predict the status of the remaining lymph nodes (Kim et al. 2006). However, several issues have been reported with use of SLNB for nodal staging. First, it was reported that the proportion of patients with successfully mapped SLNs ranged from 41 to 100 %, with >50 % of studies reporting a rate <90 %. The false-negative rate ranged from 0 to 29 %, with an average rate of 7.3 % (Kim et al. 2006). Second, patients may experience local disease recurrence following SLNB. Axillary local recurrence rates in patients with a negative SLNB and no ALND were reported to range from 0 to 1.4 % at 14–46 months of follow-up (Naik et al. 2004).

Generally, the combined use of blue dye and isotope is recommended for success and accuracy of SLNB (Albertini et al. 1996; Cody et al. 2001; Cox et al. 1998; Noguchi et al. 2000). Goyal et al. reported that in approximately 4 % of patients the positive SLN was found by dye alone and in 3 % by isotope alone; these would have been missed by relying on a single technique of localization (Goyal et al. 2006). However, radioisotope method has several problems; firstly, the scintigrams cannot clearly visualize the direct connection between primary SLNs and their afferent lymphatic vessels. Secondly, the radioisotope method requires expensive equipment and facilities. Johnson et al. reported that the radioisotope method costed $1257.06 for one patient (Johnson et al. 2011). Thirdly, there is what little radiation exposure for both patients and medical staffs (Kimura et al. 2013).

For the reasons described above, Dye-only methods have been widely performed in the world without nuclear medicine departments. This method requires a high level of technical skill to trace the dye-stained lymphatic route to SLNs. It is, therefore, very important to preoperatively detect both true SLNs and the afferent lymphatic route from the tumor and areola.

Omoto et al. reported a SLN detection method using CEUS with Sonazoid injected subareolarly in breast cancer patients. CEUS with Sonazoid method requires only a US apparatus and a contrast agent readily available on market and does not need radioactive materials. The greatest merits of this method are that SLNs can be identified without exposing the patient to radioactivity, in real-time, with visual images (Omoto et al. 2009). In our study, All the SLNs were identified using CEUS method with Sonazoid. Moreover, the cost of CEUS with Sonazoid method was low, only $162.42 for one patient. These results suggested that the combination of CEUS with Sonazoid method and blue dye method is useful for the detection of SLN and could take the place of the standard combination of isotope and blue dye methods.

Axillary lymph node metastasis is a key factor for the prognosis of breast cancer and has a major impact on decisions regarding treatment modalities; thus, diagnostically accurate methods for determining axillary lymph node metastasis are very important to make a correct staging for breast cancer patients. Axillary US is widely used for the detection of axillary lymph node metastasis because it is relatively accurate and noninvasive. Axillary US is simple, easy, and less expensive than other modalities. Therefore, it is an elemental test in breast cancer evaluation. The sensitivity and specificity of axillary US for the detection of axillary lymph node metastasis are 61.4 and 82.0 %, respectively (Houssami et al. 2011). In spite of presenting a high accuracy in many studies, the diagnostic criteria for malignancy and the indication of the method remain controversial. The vascularization studied at Doppler ultrasonography, basically follows two patterns, namely the central pattern, with a single hilum vascular signal or dispersed signals distributed at the center of the organ, and the peripheral pattern, where a linear signal is observed along the peripheral zone of the organ. Peripheral vascularization is more frequently found in metastatic lymph nodes, while the central pattern is more frequently found in the absence of malignancy. The importance of the utilization of Doppler ultrasonography as a diagnostic criteria is observed as it is associated with other morphological characteristics and not as an isolated criteria (Pinheiro et al. 2014). Using color Doppler US increases the information about lymph node, but that is limited to the macrovessel vascularity. Contrast-enhanced magnetic resonance imaging (cMRI) is generally used to evaluate the regional extent of breast cancer before breast-conserving surgery; it enables the examination of changes in the extent of tumor growth pre- and post-chemotherapy, screening of high-risk patients and those with large breasts, evaluating isolated axillary lymph node metastasis of unknown origin, and evaluating axillary lymph node metastasis in breast cancer (Ko et al. 2007; Mameri et al. 2008). The sensitivity and specificity of cMRI for the prediction of axillary lymph node metastasis range from 36 to 100 % and 54 to 100 %, respectively. These ranges are fairly wide because they are dependent on the definition of axillary lymph node metastasis, the type of contrast agent used, the size of the breast tumor, and the number of metastatic axillary lymph nodes (Garcia Fernandez et al. 2011; Harnan et al. 2011; Kvistad et al. 2000; Peare et al. 2010; Valente et al. 2012). Hwang et al. reported that the actual accuracy of cMRI was similar to that of axillary US (Hwang et al. 2013). Imaging with fluoro-2-deoxy-d-glucose-positron emission tomography (PET) is also used to evaluate axillary lymph node metastasis. The fundamental strength of PET imaging over conventional imaging is its ability to convey functional information that even the most exquisitely detailed anatomic image cannot provide. However, when surveyed across the multitude of prior reports, PET has an overall sensitivity of 88 %, specificity of 92 %, and accuracy of 89 %; however, several of the studies achieved higher sensitivity at the expense of lower specificity or vice versa. This has led to a wide variation in results (Quon and Gambhir 2005).

Sonazoid, a new generation contrast agent for ultrasonography, was first manufactured on January 10, 2007 and is approved for use only in Japan. The active ingredient of Sonazoid is a perflubutane microbubble that is stabilized using hydrogenated egg phosphatidyl serine sodium, which is a phospholipid. Perflubutane is chemically stable and insoluble in water. Therefore, it has a long lifespan in the body because it hardly dissolves in the blood. CEUS with Sonazoid administering intravenously for liver tumors has come to be frequently performed in Japan. Aoki et al. suggested that CEUS with Sonazoid administering intravenously is useful in distinguishing tumor-induced and inflammation-induced lymph node enlargement (Omoto et al. 2009; Aoki et al. 2011).

CEUS with Sonazoid administering intravenously can also allow for the visualization of microvessels. Color Doppler US provides information about macrovessel flow and morphology and can evaluate palpable lymph nodes more accurately than axillary US; however, it cannot be used to evaluate microvessels and is therefore not applicable to the evaluation of nonpalpable nodes (Yang et al. 2000). We reported a breast cancer patient after SLNB whose lymph node swelling was diagnosed as no metastasis accurately using CEUS with Sonazoid administering intravenously (Matsuzawa et al. 2015). One of the advantage of CEUS with Sonazoid administering intravenously is that the operators can get clear imagination of the placement, depth and characteristics by performing this by themselves. Li et al. reported that it is important to check blood vessel volume and density for evaluation of lymph node metastasis in vivo system (Li et al. 2013).

There were three misdiagnosed cases by using CEUS with Sonazoid administering intravenously those included; one false positive case and two false negative cases (Table 2). In false positive case No. 1, multiple microvessels were visualized by using CEUS with Sonazoid administering intravenously. However, the pathological findings revealed that there was the flow from only hilum and negative metastasis. In false negative case No. 1, the blood flow from only hilum was detected by CEUS with Sonazoid administering intravenously, but the result of pathological findings revealed the micro invasion less than 1 mm. In false negative case No. 2, it was difficult to visualize the hilum in itself. These problems will be solved by improvement of the ultrasound apparatus, accumulation of cases and our knowledge for the imaging features of SLNs of positive or negative metastasis. In the present study, CEUS with Sonazoid administering intravenously was the most accurate evaluation compared with other modalities. By overcoming these problems described above, we propose that the combination of CEUS with Sonazoid administering both subareolarly and intravenously can result in an accurate diagnosis of axillary lymph node metastasis.

**Table 2:** Clinical and pathological characteristics of misdiagnosed cases by using CEUS with Sonazoid administering intravenously

	Age	Surgery	pT	pN	pStage	Histology	CEUS with Sonazoid	Pathological findings of SLN	Cause of misdiagnosis in CEUS with Sonazoid
False positive case no. 1	40	BCS + SLNB	pT1c	N0	IA	MUC	Positive metastasis	Negative metastasis	Multiple microvessels
False negative case no. 1	51	MRM + SLNB + Ax	pT1c	N1mi	IB	IDC	Negative metastasis	Positive metastasis	Detection of flow from only hilum
False negative case no. 2	77	MRM + SLNB + Ax	pT2	N1	IIIIA	IDC	Negative metastasis	Positive metastasis	Difficulty of visualization of hilum

## Methods

### Patients

This study was approved by the Bioethics Committee of Hokkaido social work association Obihiro Hospital. The clinical study protocol was explained in detail to patients eligible for the study. If a patient agreed to participate, she would then sign an informed consent form and was registered for the study.

Between December 2013 and May 2014, 32 female patients (median age 60.4 years, range 32–86 years) with histologically diagnosed as breast cancer by US guided core needle biopsy. It was confirmed that they did not have distant metastases by contrast enhanced computed tomography (CECT) findings. The clinical and pathological characteristics are shown in Table 3. The patients included 6 cases (18.6 %) in pTis stage, 5 cases (15.6 %) in pT1b stage, 9 cases (28.1 %) in pT1c stage, 3 cases (9.4 %) in ypT1c stage, 7 cases (21.9 %) in pT2 stage and 2 cases (6.4 %) in ypT2 stage. The patients included 21 cases (65.6 %) in pN0 stage, 1 cases (3.1 %) in pN1mi stage and 10 cases (31.3 %) in pN1 stage. 9 cases (28.1 %) of patients were performed breast conserving surgery and 23 cases (71.9 %) of patients were performed modified radical mastectomy. 19 cases (59.4 %) of patients were performed SLNB and no metastasis in SLN, but 9 cases (28.1 %) of patients were positive SLN metastasis so underwent axillary dissection in addition. Four cases (12.5 %) of patients were considered to have positive metastasis in the axillary lymph nodes so they underwent axillary dissection from the beginning.

**Table 3:** Clinical and pathological characteristics of 33 patients with breast cancer

	No. of patients	Percentage
Age (years)	60.4 ± 13.2 (range 32–86)	
Palpability		
Breast tumor		
Yes	22	68.8
No	10	31.2
ALN		
Yes	2	6.3
No	30	93.7
Surgery		
Breast		
BCS	9	28.1
MRM	23	71.9
Axilla		
SLNB	19	59.4
SLNB + AD	9	28.1
AD	4	12.5
Laterality		
Right	11	34.4
Left	21	65.6
Tumor size (mm)	21.9 ± 19.2 (6–115)	
Multiplicity		
Yes	4	12.5
No	28	87.5
T stage		
pTis	6	18.6
pT1a	0	0
pT1b	5	15.6
pT1c	9	28.1
ypT1c	3	9.4
pT2	7	21.9
ypT2	2	6.4
N stage		
0	21	65.6
1mi	1	3.1
1	10	31.3
Stage		
0	6	18.8
IA	11	34.4
IB	1	3.1
IIA	7	21.9
IIB	3	9.4
IIIA	4	1.3
Histology		
IDC	23	71.9
DCIS	5	15.6
MUC	3	9.4
SCC	1	3.1

*BCS* breast conserving surgery, *MRM* modified radical mastectomy, *SLNB* sentinel lymph node biopsy, *AD* axillary dissection, *IDC* invasive ductal carcinoma, *DCIS* ductal carcinoma in situ, *MUC* mucinous carcinoma, *SCC* squamous cell carcinoma

### Imaging examinations

All patients underwent CECT for the evaluations of systemic metastasis including ALNs before admission.

**Fig. 3:**
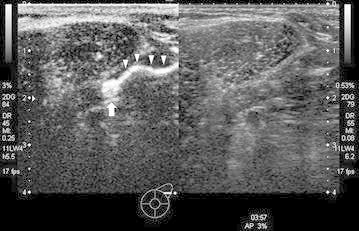
An Axillary lymph node detection using contrast-enhanced ultrasonography with Sonazoid injected subareolarly. A single route/single SLN pattern in a 64-year-old woman with 18 × 17-mm tumor in the left lower outer quadrant area, where only a single common lymph vessel from the subareolar area (*white arrow head*) drains into a single common SLN (*white arrow*)

In the morning on the day before the operation, at first the patients underwent conventional US (including B-mode and color Doppler) for re-check the metastasis of ALNs. Next, injections of 1 ml of Sonazoid were gently administered using a plastic syringe with a 22G-needle into subareolar region. The transducer was placed lightly on the skin and the area from the outside of the upper lateral quadrant area to the axillary region was observed by US. The long axial view was scanned parallel to a line connecting the nipple and the axilla. The ultrasound equipment used in this study was an Aplio500 (Toshiba medical systems, Tokyo, Japan). Contrast enhanced scanning was performed using coded-phase inversion harmonic US with mechanical indices (MIs) of 0.15–0.19, of 10–20 mm from the surface (Fig. 3). We marked the skin directly above the detected SLN. In the evening on the same day, the patients underwent CEUS by an administration intravenously with Sonazoid (0.015 ml/kg body weight). If the contrast-enhancement of SLNs was remained before intravenous injection, we pointed the ultrasound beam to the SLNs for destroying the bubble of Sonazoid using conventional B-mode scan with high MI until the disappearance of the enhancement. We used the same Aplio500 as the SLN detection for this examination.

After the CEUS with Sonazoid injected subareolarly, the patients underwent CT lymphography for the detection of SLNs. CT lymphography was performed using a 64-detector row CT scanner (Toshiba, Aquilion 64, Japan). Under local anesthesia, 3 ml of undiluted iopamidol (Iopamiron 370; Bayer, Osaka, Japan) was injected into the periareolar areas followed by gentle massage for 60 s. Each patient was placed in the supine position with arms positioned in a cranial direction. We marked the skin directly above the detected SLN using another color.

In the operation while patients were under general anesthesia, a 5 ml of indigo carmine blue dye was injected into the periareolar areas, followed by 1 min of massage to promote lymphatic flow. An incision (2–3 cm) was made along the skin surface corresponding to the marks that had been made during CEUS with Sonazoid administering subareolarly and CT lymphography. Then SLNs were identified and biopsied during breast-conserving surgery. In case involving mastectomy, we attempted to identify SLNs after making a skin flap. We tried to detect SLNs by following the lymphatic route dyed by indigo carmine. The entirety of the SLN was cut along the longitudinal axis into sections of 2.0 mm thickness. When a positive SLN was found in a frozen section, total axillary lymph node dissection (ALND) was performed.

### Identification of SLNs by CEUS with Sonazoid injected subareolarly

We compared the location which was detected preoperatively by CEUS with Sonazoid administering subareolarly and CT lymphography with the location that was detected intraoperatively by indigo carmine method. The correspondence ratio between these data was analyzed.

### Evaluation of axillary lymph node metastasis in SLNs by using CECT

Metastatic lymph nodes were evaluated independently by two breast surgeon (F.M., T.E.) who did not know the clinical outcome based on the short-axis diameter (>5 mm), internal fat density indicating absence of a central image, and early strong enhancement, compared with the late phase of the ALNs on CT images.

### Evaluation of axillary lymph node metastasis in SLNs by using color Doppler US and CEUS with Sonazoid administering intravenously

Three breast surgeons (F.M., T.E., T.S.) and ultrasonographers performed US of axilla as written bellow. Lymph nodes were categorized as positive metastasis if they exhibited more than two number of vascularities except for the place of hilum.

### Histopathological examinations

Post operatively, all slices were fixed in 10 % buffered formalin, embedded in paraffin, and microscopically examined using hematoxylin and eosin staining. Hematoxylin-eosin stained tumor deposits not larger than 2 mm were defined as micrometastasis.

### Statistical analysis

We calculated the sensitivity, specificity, positive predictive value (PPV), negative predictive value (NPV), and accuracy (ACC) for color Doppler US, CECT and CEUS with Sonazoid comparing the imaging findings with pathological results. We used χ^2^ test or Fisher exact test to determine the correlation among these results. All differences were considered significant P < 0.05. All statistical analysis were performed using JMP pro 11 software (SAS Institute Japan).

## Conclusion

This study revealed that the evaluation of axillary SLN detection using CEUS with Sonaziod administering subareolarly and the evaluation of metastasis in SLNs using CEUS with Sonazoid administering intravenously is the most accurate in those using other modalities, the accuracy reached over 90 %. CEUS with Sonazoid can evaluate microvessels, and that is the cause of improved results. Although more experiences are needed for further improvement of accurate diagnosis, the evaluation of axillary SLN metastasis using combination of CEUS with Sonazoid administering both subareolarly and intravenously would take the place of conventional sentinel lymph node biopsy in breast cancer surgery.

## Abbreviations

SLNB: sentinel lymph node biopsy
SLNs: sentinel lymph nodes
ALND: axillary lymph node dissection
ALNs: axillary lymph nodes
CEUS: contrast enhanced ultrasonography
US: ultrasonography
CECT: contrast enhanced computed tomography
MIs: mechanical indices
PPV: positive predictive value
NPV: negative predictive value
ACC: accuracy
cMRI: contrast-enhanced magnetic resonance imaging
PET: fluoro-2-deoxy-d-glucose-positron emission tomography
